# IL-7 and CD4 T Follicular Helper Cells in HIV-1 Infection

**DOI:** 10.3389/fimmu.2017.00451

**Published:** 2017-04-20

**Authors:** Francesca Chiodi, Yonas Bekele, Rebecka Lantto Graham, Aikaterini Nasi

**Affiliations:** ^1^Department of Microbiology, Tumor and Cell Biology, Karolinska Institutet, Stockholm, Sweden

**Keywords:** T follicular helper cells, IL-7, CD127, costimulatory molecules, memory B cells

## Abstract

IL-7 was previously shown to upregulate the expression of molecules important for interaction of CD4+ T cells with B cells. It is poorly studied whether IL-7 has a role in the biology of T follicular helper (Tfh) cells and whether IL-7 dysregulates the expression of B-cell costimulatory molecules on Tfh cells. We review the literature and provide arguments in favor of IL-7 being involved in the biology of human Tfh cells. The CD127 IL-7 receptor is expressed on circulating Tfh and non-Tfh cells, and we show that IL-7, but not IL-6 or IL-21, upregulates the expression of CD70 and PD-1 on these cells. We conclude that IL-7, a cytokine whose level is elevated during HIV-1 infection, may have a role in increased expression of B cell costimulatory molecules on Tfh cells and lead to abnormal B cell differentiation.

## Introduction

T follicular helper (Tfh) cells play a pivotal role in the generation of long-lived humoral immunity by providing help to B cells in the germinal centers (GCs), ensuring their differentiation to memory B cells and plasma cells (PCs). During the last decade, the differentiation program of Tfh has been unraveled and molecular pathways involved in this process have been identified.

The maturation phase of Tfh cells comprises three distinct, interconnected steps. The process begins in the secondary lymphoid organs once CD4+ T cells interact with antigen-loaded dendritic cells (DCs) (1). While all molecules involved in the decision of redirecting naïve CD4+ T cells into the Tfh cell lineage are not identified, it is shown that this switch occurs already after a few divisions of activated CD4+ T cells (2, 3). The upregulation of Bcl-6 expression on committed Tfh cells coincides with upregulation of CXCR5 chemokine receptor expression, whereas the expression of CCR7, on the contrary, declines (2). On CXCR5 upregulation, Tfh cells migrate into B cell follicles where they complete their differentiation process by downmodulating the Bcl-6 protein (3).

## Cytokines with Known Involvement in the Differentiation Process of Tfh Cells in Humans

The differentiation of Tfh cells relies on signaling pathways and transcription factors, which are activated in response to selected cytokines. Three cytokines, as demonstrated in multiple studies, control the differentiation of Tfh cells in humans: IL-12, IL-23, and transforming growth factor-beta (TGF-β). Activated DCs, through IL-12 production, promote the differentiation of naïve CD4+ T cells into IL-21-producing Tfh-like cells (4), a process that is primarily dependent on STAT4. IL-23, a cytokine that is also produced by activated DCs, acts in synergy with IL-12 to promote the phenotypical and transcriptional shift of naive CD4+ T cells into Tfh cells (5). The process of Tfh differentiation is further supported by the immunomodulatory cytokine TGF-β, which enhances the function of STAT3 and STAT4 (6) and induces downregulation of Blimp-1 in CD4+ T cells activated by DCs, a process that leads to a Tfh cell Blimp-1^neg^Bcl-6^pos^ phenotype.

HIV-1 infection is characterized by the dysregulated expression of multiple cytokines. A detailed picture on whether HIV-1 infection in humans affects the production of cytokines involved in differentiation of Tfh cells is yet not available. In a recent study, TGF-β and the active form of IL-12 were measured in the plasma of HIV-1-infected patients with clinical progressive and non-progressive HIV-1 infection (7). The level of TGF-β was higher in both groups of patients when compared to controls and was not affected by the length of antiretroviral treatment (ART); IL-12 concentration in plasma was higher in patients with non-progressive HIV-1 infection compared to controls. TGF-β concentration was significantly decreased during non-progressive HIV-1 infection compared to progressive infection, whereas the opposite pattern was noticed for the active form of IL-12 (7).

Activation of peripheral blood mononuclear cells (PBMCs) from HIV-1-infected patients leads to reduced IL-12 production (8), and the impaired capacity of DCs from HIV-1-infected subjects to produce IL-12 *ex vivo* does not improve upon ART (9).

## IL-7 and Expression of IL-7 Receptor Alpha (CD127) During HIV-1 Infection

IL-7 is an essential survival factor for resting naive and memory T cells; knowledge on whether IL-7 has a role in differentiation or maintenance of Tfh cells is limited. An increased serum IL-7 concentration was reported during HIV-1 infection [reviewed in Ref. (10)], suggesting an altered availability of this cytokine at various sites. Multiple sources of IL-7 have been described, including keratinocytes, fibroblasts, bone marrow stromal cells, thymic epithelial cells, the intestinal epithelium, and DCs (10). The lymphoid tissue reticular fibroblast network was also identified as a major source of IL-7 for T cells residing in secondary lymphoid tissues (11). High serum IL-7 levels were mostly observed in lymphopenic patients likely resulting from reduced IL-7 consumption following T cell depletion.

Two recent studies indicated that IL-7 might strongly influence the biology of murine Tfh cells. During mouse lymphocytic choriomeningitis virus infection, Tfh memory cell precursors were characterized by an early expression of CD127, which distinguished Tfh cells from Bcl-6^neg^ activated T cells (12). In addition, specific influenza vaccine antibody responses were efficiently boosted by IL-7, which acted by increasing Tfh cell frequency in lymph nodes (13); this IL-7 effect was specific for Tfh cells and did not affect other types of T helper cells. These recent findings suggest that IL-7 in mice may influence both the generation and maintenance of Tfh cells; in addition, this cytokine may be useful to induce selected clones of Tfh cells upon vaccination, thus enhancing protective humoral responses. The role of IL-7 in the biology of Tfh cells is, however, still controversial as it was shown that IL-7 signaling represses the expression of the Tfh-associated gene Bcl-6 through STAT5 activation (14). Moreover, the expression of CD127 was low within GC Tfh cells of macaques studied in the context of SIV vaccination, but relatively higher in CD4+CXCR5+PD-1+ T cells in lymph nodes (15). It is possible that differences in CD127 expression on Tfh cells reported in different studies may reflect distinct stages of Tfh cell differentiation, a process that is highly complex and dynamic.

An expansion of Tfh cells in HIV-1-infected subjects that positively correlated to the frequency of GC B cells (16) has been reported; the mechanism for this expansion of Tfh cells is yet unknown. A memory subset of Tfh cells related to Tfh cells resident in lymph nodes and characterized by CXCR5 expression was shown to circulate in blood (17, 18). A recent study indicated that circulating IL-21+CD4+ T cells may be an accurate counterpart of Tfh cells resident in lymphoid tissue, as determined by functional, phenotypical, and transcriptional characteristics (19).

**Figure 1:**
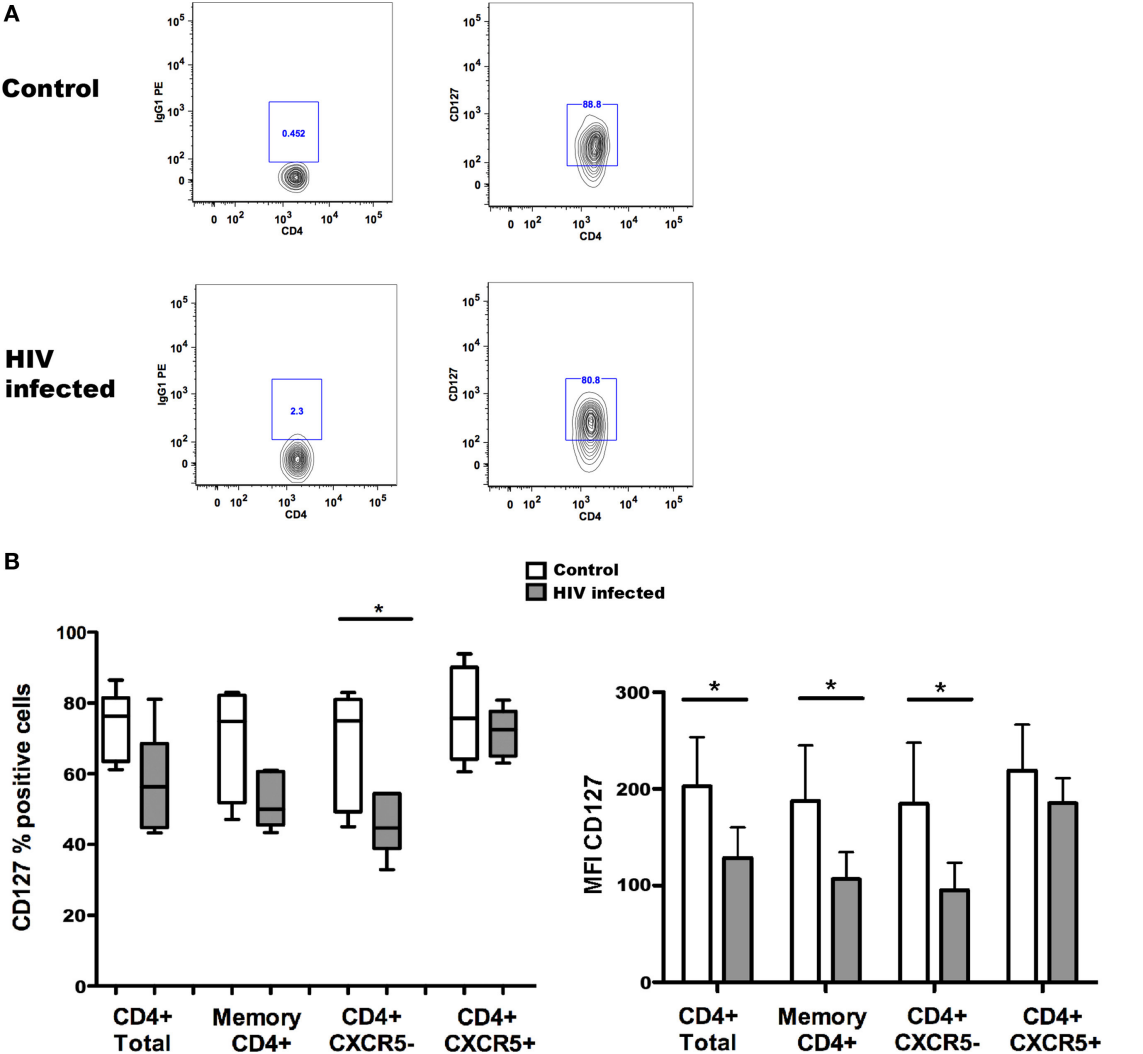
**CD127 expression on memory T follicular helper (Tfh) cells from controls and HIV-1-infected individuals**. The frequency of CD127+ cells [**(B)** left] and CD127 mean fluorescence intensity (MFI) [**(B)** right] were determined among total CD4+ and memory CD4+ T cells and Tfh (CXCR5+) and non-Tfh (CXCR5−) cells from non-infected control subjects and HIV-1-infected patients receiving antiretroviral treatment (ART). Representative flow cytometry plots of CD127 expression in one control and one HIV-1-infected individual **(A)**; the cells were gated on alive, CD4+CD45RO+CXCR5+ Tfh cells before evaluation of CD127 expression. Blood samples were obtained from six healthy donors and six HIV-1-infected patients receiving ART, characterized by undetectable viremia (<20 copies/ml) and a mean CD4+ T cell count of 588 ± 443 cells/μl. Peripheral blood mononuclear cells were isolated using Ficoll gradient centrifugation (Lymphoprep, Axis-Shield PoC As, Norway) and stored frozen until required. The following fluorochrome-conjugated anti-human antibodies were used to characterize T cell populations: V500 and PE-anti-CD4, FITC anti-CD45RO, PerCP-Cy5.5, and Alexa Fluor 647 anti-CXCR5 and PE anti-CD127 (BD Pharmingen, San Diego, CA, USA). Viable cells were detected using Near-IR-Live/Dead (ViViD) kit (Invitrogen, Carlsbad, CA, USA). Fluorescence intensities were measured with LSRII (BD, San Jose CA, USA), and data were analyzed using FlowJo version 8.8.7 (Tree Star Inc., Ashland, OR, USA). Memory CD4+ T cells (CD4+ and CD45RO+) were further gated into Tfh (CXCR5+) and non-Tfh (CXCR5−) cell subsets based on the CXCR5 expression. Data were tested for normal distribution by Kolmogorov–Smirnow test, and analyses were performed using non-parametric Mann–Whitney test when comparing values from HIV-1-infected patients with non-infected controls. Statistical analyses were performed using Prism (version 5.0a, GraphPad Software Inc., San Diego, CA, USA). Data are represented by box and Whisker plots (minimum to maximum values). *p < 0.05.

Taking advantage of the possibility of studying CXCR5+ Tfh cells in blood, we assessed the expression of CD127 on circulating memory Tfh cells in healthy controls and HIV-1-infected individuals. The results of these experiments are illustrated in Figure 1. The expression of CD127 was analyzed on total and memory CD4+ T cells, Tfh cells characterized as CD4+CD45RO+CXCR5+, and their counterpart non-Tfh-cells CD4+CD45RO+CXCR5−; all these populations were found to be CD127 positive in blood from healthy controls. The frequency of CD127+ cells was slightly reduced among all T cell subpopulations of HIV-1-infected individuals (Figure 1) reaching a significant difference only for CD4+CXCR5− cells. In addition, the CD127 mean fluorescence intensity (MFI) was reduced on different T cell subpopulations obtained from HIV-1-infected patients when compared to controls (Figure 1). It was previously shown that expression of CD127 is lost on a large proportion of peripheral T cells, both CD4+ and CD8+, in HIV-1-infected patients presenting with lymphopenia (20, 21); this feature of HIV-1 immunopathology is ameliorated by ART introduction. The results presented here show that circulating Tfh cells and non-Tfh cells express CD127 and therefore may be potential IL-7 targets.

Tfh cells, defined as CXCR5+PD-1+Bcl-6+, in lymph nodes of HIV-1-infected patients were shown to comprise the highest percentage of CD4+ T cells harboring HIV-1 DNA (22). Peripheral Tfh cells from HIV-1-negative individuals are highly permissive to HIV-1 infection; the same cell type isolated from the blood of HIV-1-infected, virologically suppressed patients was shown to be a putative HIV-1 reservoir (23). Productive SIV infection within the B cell follicles of SIV-infected monkeys clinically defined as elite controllers strengthen the possibility that Tfh cells in lymph nodes represent reservoirs for SIV infection, which are not accessible to potent antiviral CD8+ T cell responses (24). Interestingly a transient blip of HIV-1 replication was monitored in patients treated with recombinant human IL-7 (25, 26). As we show that circulating Tfh cells express CD127, both in HIV-1-infected and non-infected individuals, it is possible that HIV-1 replication detected during treatment with recombinant human IL-7 may be due to IL-7-induced HIV-1 reactivation in Tfh cells, as previously reported for CD4+ T cells (27).

## Regulation of ICOS, PD-1, and CD70 Expression on Tfh Cells by IL-7

Abnormal B-cell activation, characterized *in vivo* by increased serum IgG levels in infected patients, is an important component of HIV-1 pathogenesis [reviewed in Ref. (28)]; this feature of HIV-1 infection is puzzling considering that the levels of specific antibodies against vaccination antigens are reduced in HIV-1-infected patients [reviewed in Ref. (29)].

We have previously reported that IL-7 triggers a non-antigen-specific plasmablast differentiation and antibody production in resting memory B cells through upregulation of CD70 on T cells (30). Here, we investigated whether incubation of Tfh cells, sorted from the blood of healthy controls, with IL-7 had any effect on the expression of ICOS and PD-1 molecules, which are very important for Tfh cell interaction with B cells within the GC, and of the costimulatory molecule CD70.

ICOS expression may be required during the early steps of Tfh cell differentiation for Bcl-6 induction in naïve CD4+ T cells in response to ICOS-L provided by activated DCs (31). ICOS on Tfh cells is an important costimulatory receptor for T-dependent antibody responses in GCs, as demonstrated by the consistent reductions of antibody responses and memory B cells in ICOS-deficient individuals (32, 33). The absence of ICOS expression is also associated with a reduced number of Tfh cells.

PD-1, generally viewed as an exhaustion marker, is increasingly recognized as a pivotal molecule providing help for B cell differentiation. A high level of PD-1 expression on CD4+ T cells in lymph nodes of macaques was identified as a valuable marker of Tfh cells (34). The interaction of PD-1 expressed on Tfh cells and PD-L2 expressed on B cells regulates PC survival in mice (35); thus, PD-1 expressed on Tfh cells promotes humoral response and, in this context, does not appear to have the inhibitory role described in pathogenic antiviral responses. The common gamma-chain cytokines IL-2, IL-7, and IL-15 have previously been shown to induce the expression of PD-1 on T cells and of PD-L1 and PD-L2 on macrophages (36).

The CD70 costimulatory molecules expressed on activated lymphocytes and DCs can increase activation of B and T cells through binding to CD27 (37). CD27-mediated cell activation is regulated by the transient nature of CD70 expression, and continuous expression of this molecule has deleterious effects; in fact, in mice constitutively expressing CD70 on B cells, T cells are under continuous immune activation leading to severe immunodeficiency characterized by depletion of naive T and B cells (38, 39). We also showed that during HIV-1 infection, the high frequency of circulating CD4+CD70+ T lymphocytes in HIV-1-infected individuals was associated with increased plasma IgG levels, upregulation of the B cell activation marker CD38 on memory B cells, and proliferation of memory B cells (40). Thus, CD4+CD70+ T lymphocytes appear to have a role in increased B cell activation during HIV-1 infection [reviewed in Ref. (28)].

**Figure 2:**
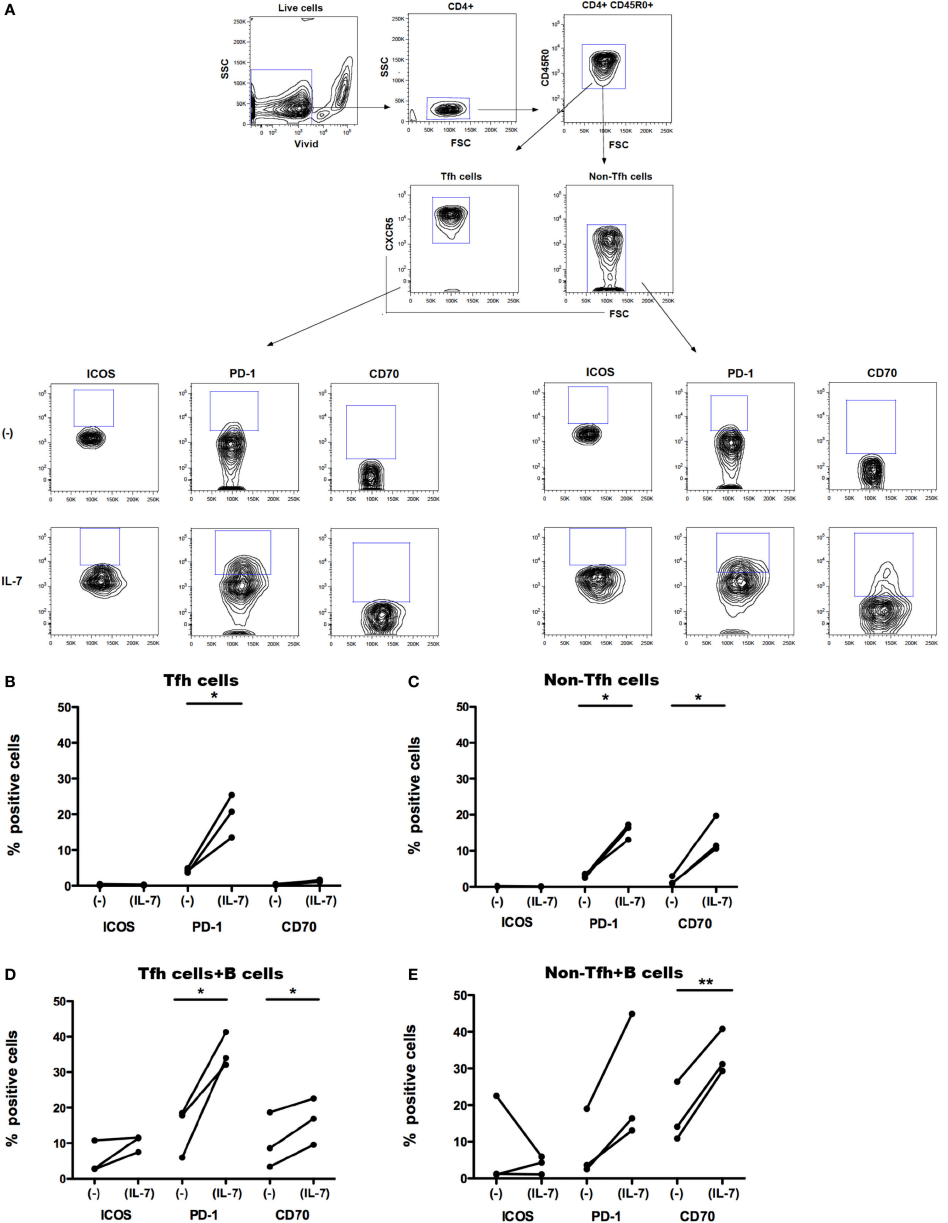
**Effect of IL-7 and B cell coculture on ICOS, PD-1, and CD70 expression on blood T follicular helper (Tfh) and non-Tfh cells**. The gating strategy for identification of Tfh cells in blood **(A)**. Small CD4+ cells were identified by CD4 expression. Aggregates and dead cells were excluded by subsequent altered forward light scatter height–width ratios and dead cell dye uptake, respectively. Gated live memory CD4+ T (CD4+CD45R0+) single cells were then evaluated to identify Tfh cells (CD4+CD45R0+CXCR5+) and non-Tfh cells (CD4+CD45R0+CXCR5−). The frequency of ICOS+, PD-1+, and CD70+ cells was thereafter determined in Tfh and non-Tfh cells cultured in the presence and absence of IL-7. ICOS, PD-1, and CD70 expression on freshly sorted Tfh (CXCR5+) **(B)** and non-Tfh (CXCR5−) cells **(C)** cultured in the presence or absence of IL-7 for 6 days is shown. Frequency of ICOS, PD-1, and CD70 was also measured on Tfh **(D)** and non-Tfh cells **(E)** cocultured with B cells alone or with B cells in the presence of IL-7 for additional 7 days. Results of three independent experiments are shown. *p < 0.05, **p < 0.01. Freshly isolated peripheral blood mononuclear cells obtained from healthy controls (buffy coats) were processed to isolate CD4+ T cells by negative selection and stained with ViViD, PE-anti-CD4, FITC anti-CD45RO, and Alexa Fluor 647 anti-CXCR5 before sorting on a BD FACS Aria. Memory Tfh (CXCR5+) and non-Tfh (CXCR5−) cells were obtained at purities of >99%. Isolated Tfh and non-Tfh cells were washed and resuspended in RPMI medium supplemented with 10% fetal calf serum with or without IL-7 (25 ng/ml) for 6 days and cocultured with B cells for additional 7 days; the incubation times were selected according to previous experiments for optimal modulation of CD70 expression on T cells treated with IL-7 (30). For coculture of B cells with Tfh and non-Tfh cells, B cells were separated by negative selection using B cells isolation kit II (Miltenyi Biotech, Bergish Gladbach, Germany). Recombinant human IL-7 (Peprotech, London, UK) was used at a concentration of 25 ng/ml. The following fluorochrome-conjugated anti-human antibodies were used: V500 and PE-anti-CD4, FITC anti-CD45RO, PerCP-Cy5.5 and Alexa Fluor 647 anti-CXCR5, BV-421 anti-PD-1 (CD279), PE anti-ICOS (CD278), PE-Texas Red, and PE anti-CD70 (BD Pharmingen, San Diego, CA, USA). Viable cells were detected using Near-IR-Live/Dead (ViViD) kit (Invitrogen, Carlsbad, CA, USA). Fluorescence intensities were measured with LSRII (BD, San Jose, CA, USA), and data were analyzed using FlowJo version 8.8.7 (Tree Star Inc., Ashland, OR, USA). Wilcoxon matched pair *t*-test was used to compare the effect of IL-7 treatment on the expression of different surface molecules on Tfh and non-Tfh cells of the same individuals. Statistical analyses were performed using Prism (version 5.0a, GraphPad Software Inc., San Diego, CA, USA). The difference in CD127 MFI was tested with Kolmogorov–Smirnov test, and analyses were performed using unpaired *t*-test.

We separated Tfh cells (CD4+CD45RO+CXCR5+) and their non-Tfh cell counterpart (CD4+CD45RO+CXCR5−) from PBMCs obtained from healthy controls. *In vitro*, CXCR5+CD4+ T cells display more pronounced properties to activate B cells compared to CXCR5−CD4+ T cells (17). Isolated Tfh and non-Tfh cells were cultured in the presence of IL-7 (for 6 days) and further cocultured with B cells (for additional 7 days). We studied whether IL-7 affects the expression of ICOS, PD-1, and CD70 on Tfh and non-Tfh cells and whether coculture of Tfh cells with B cells further modulates the expression of these molecules. The gating strategy for Tfh and non-Tfh cells and the expression of ICOS, PD-1, and CD70 on these cells are shown in Figure 2A.

**Figure 3:**
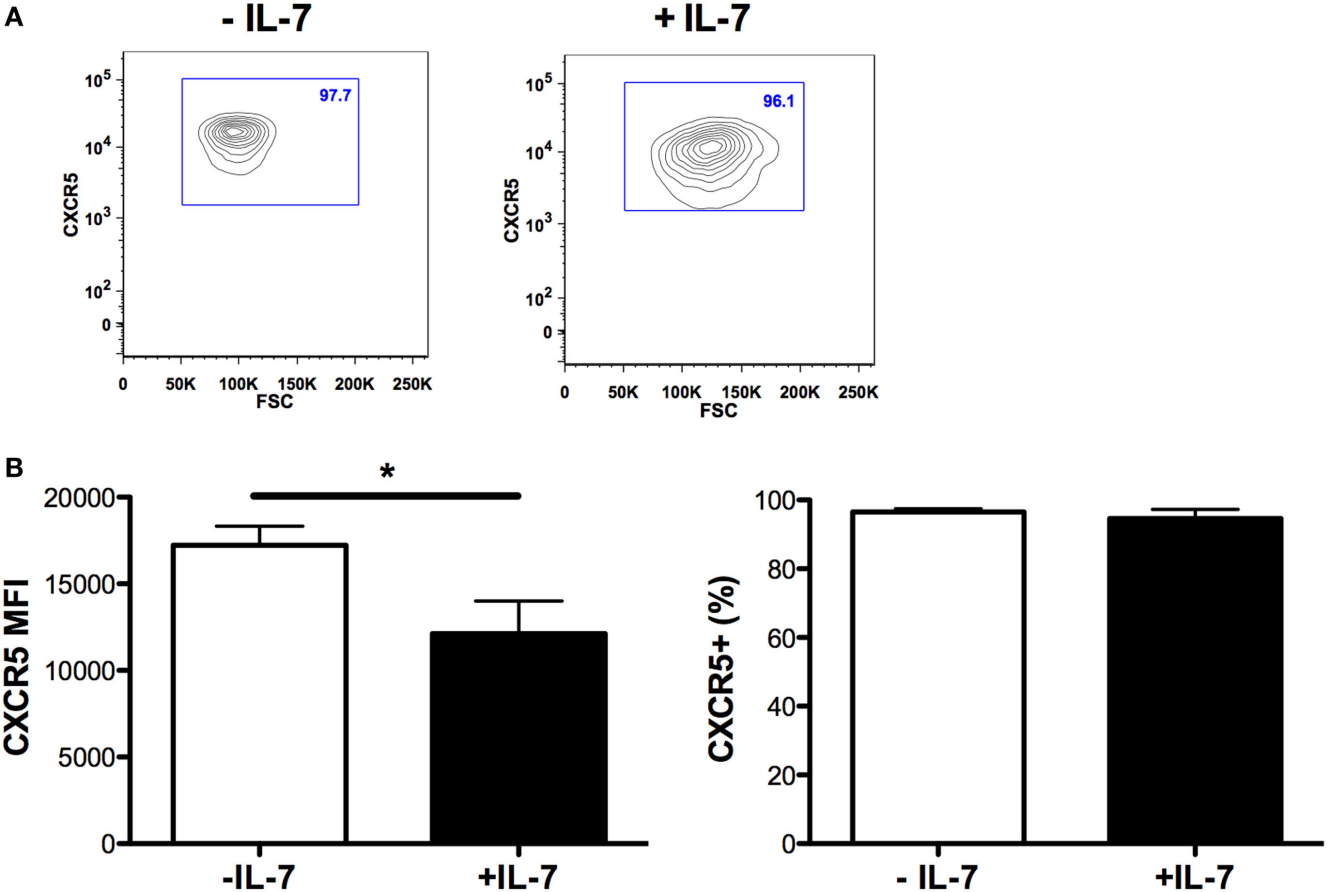
**CXCR5 expression on T follicular helper (Tfh) cells cultured in the presence of IL-7**. The mean fluorescence intensity of CXCR5 on Tfh cells [left panel **(B)**], and the frequency of CXCR5-expressing Tfh cells [right panel **(B)**] was determined in sorted Tfh cells and cultured with or without IL-7 (25 ng/ml) (Peprotech, London, UK) for 6 days. The samples were analyzed using the FlowJo version 9.9.4 (Tree Star Inc., Ashland, OR, USA). Representative flow cytometry plots showing frequency of CXCR5+-sorted Tfh cells cultured in the presence or absence of IL-7 **(A)**.

The expression of ICOS, PD-1, and CD70 was very low in Tfh and non-Tfh cells cultured in the absence of IL-7 (Figures 2B–E). IL-7 in culture significantly upregulated the expression of PD-1 on both Tfh and non-Tfh cells, whereas this cytokine had a distinct effect on CD70 expression only on non-Tfh cells. The coculture of Tfh and non-Tfh cells with B cells led to upregulated expression of PD-1 and CD70 in both cell types indicating that B cells exert a direct role in modulating the expression of molecules important for their differentiation. IL-7, when added to cocultures of Tfh and B cells, further upregulated the expression of PD-1 and CD70, whereas ICOS expression on Tfh cells was not influenced by IL-7. In coculture of non-Tfh cells with B cells in the presence of IL-7, a significant upregulation of CD70 was revealed; PD-1 expression was also increased although not to a significant level. The frequency of CXCR5+-sorted Tfh cells was not altered by the presence of IL-7 in culture, whereas the level of expression of CXCR5 on Tfh cells was slightly reduced following treatment with IL-7 (Figure 3).

**Figure 4:**
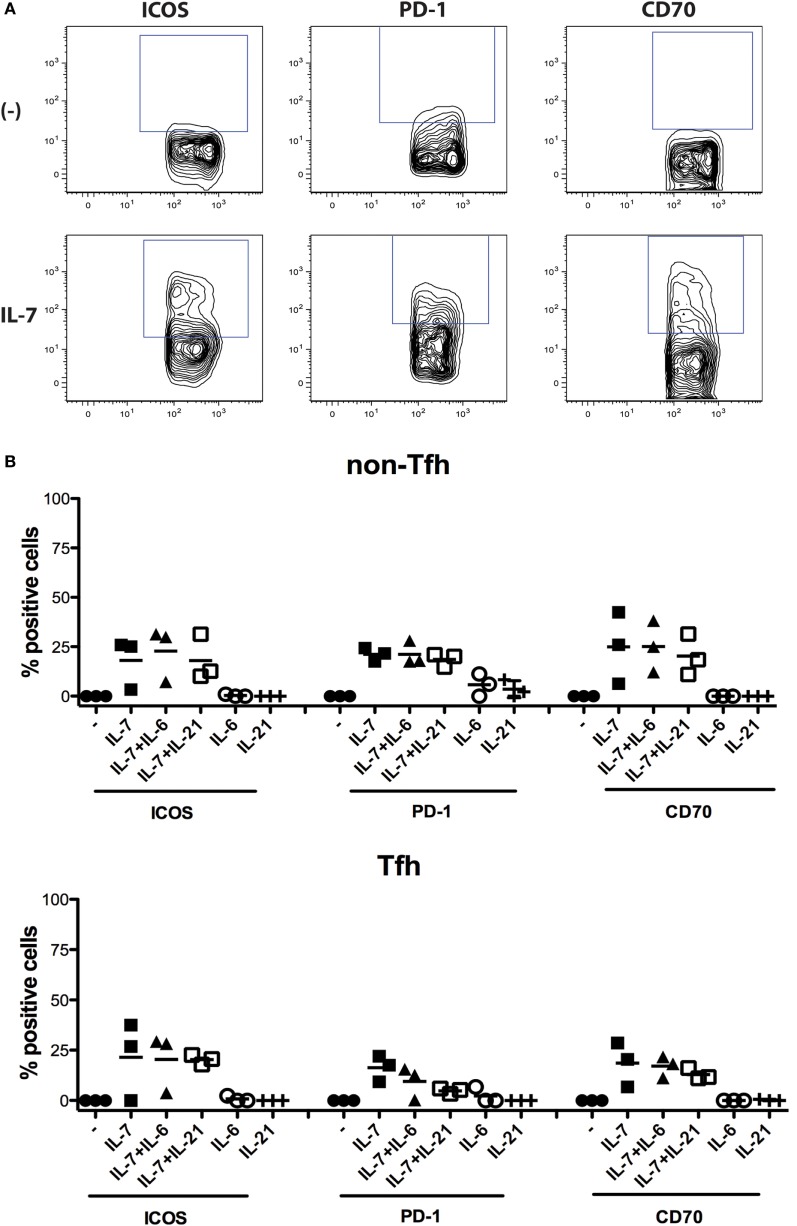
**Impact of IL-7, IL-6, and IL-21 and the combination of these cytokines on the expression of ICOS, PD-1, and CD70 on non-T follicular helper (Tfh) and Tfh cells**. Freshly isolated peripheral blood mononuclear cells from three donors were used for isolation of CD4+ T cells using the Pan T cell isolation kit from Miltenyi Biotec (Bergisch Gladbach, Germany). The CD4+ T cells were then cultured in the presence of cytokines IL-7 (25 ng/ml), IL-6 (100 ng/ml), and IL-21 (100 ng/ml) (Peprotech, London, UK), or the combination of these cytokines, for 6 days. Thereafter, the expression of ICOS, PD-1, and CD70 was measured on Tfh cells (CD4+CD45RO+CXCR5+) and non-Tfh cells (CD4+CD45RO+CXCR5−) **(B)**. The following fluorochrome-conjugated anti-human antibodies were used: V500 anti-CD4, FITC anti-CD45RO, Alexa fluor 647 anti-CXCR5, BV-421 anti-PD-1, PE CF594 anti-CD70, and PE anti-ICOS (BD Pharmingen, San Diego, CA, USA). Dead cells were excluded using the Near-IR-Live/Dead kit (Invitrogen, Carlsbad, CA, USA). The samples were analyzed using CYAN ADP analyzer (Beckman Coulter, Brea, CA, USA) and FlowJo version 9.9.4 (Tree Star Inc., Ashland, OR, USA). Representative flow cytometry plots showing the frequency of ICOS+, PD-1+, and CD70+ Tfh cells cultured in the presence or absence of IL-7 **(A)**. The effect of the different cytokines was compared both with non-parametric ANOVA and individual Wilcoxon matched pair *t*-test.

To verify whether modulation of expression of ICOS, PD-1, and CD70 by IL-7 was specific for this cytokine, we also assessed IL-21, a cardinal cytokine for several Tfh cell functions, and IL-6, as the IL-6R is highly expressed on circulating IL-21+ Tfh cells (19). The experiment revealed that the effect of IL-7 was specific (Figure 4) as IL-6 and IL-21 had no effect when used individually, and the combination of IL-7 with either IL-6 or IL-21 did not increase the level of expression of ICOS, PD-1, and CD70 to levels higher than IL-7-induced expression.

## Concluding Remarks

Very few studies have so far addressed whether IL-7 has a role in regulating the biological properties of Tfh cells. We showed here that a large proportion of circulating Tfh cells express CD127 in specimens obtained from both healthy controls and HIV-1-infected individuals. Furthermore, IL-7 in Tfh cell cultures or in cocultures of Tfh and B cells promotes the expression of PD-1 and CD70 molecules important for the interaction between Tfh and B cells.

Whether IL-7 and CD127 expressed on Tfh cells play a role *in vivo* for the interaction of these cells with B cells remains to be shown. It is possible that the confined amounts of IL-7 available upon physiological conditions may have limited consequences for the biology of Tfh cells. However, the picture may be different in diseases characterized by high levels of circulating IL-7, including chronic infections and autoimmune diseases. High IL-7 production by DCs in lymphoid tissue may lead to increased expression on Tfh cells of molecules involved in interaction with B cells.

PD-1 expression on Tfh cells was shown to have an important role for PC differentiation and survival in mice (35); it is likely that increased PD-1 expression induced by IL-7 on Tfh cells may promote an expansion of the PC pool through augmented survival. We previously showed that IL-7 triggered a non-antigen-specific plasmablast differentiation and antibody production in resting memory B cells through the upregulation of CD70 on T cells (30). It remains to be verified whether increased expression of CD70 on Tfh cells is beneficial for B cells responses *in vivo*. Previously published work (41) suggested that increased expression of CD70 on CXCR5-expressing T cells within the GCs led to a termination of B cell responses and compromised antibody production through a mechanism, which involved Fas-dependent impairment of GC B cell differentiation. Increased CD70 expression on T cells was previously detected in patients with rheumatoid arthritis (42) and systemic lupus erythematosus (43), but it is not studied whether CD70 upregulation on Tfh cells takes place during autoimmune diseases and has a role in broadening B cell responses toward autoantigens.

IL-7 was shown to be a useful adjuvant for vaccination as influenza-specific vaccine antibody responses were efficiently boosted by IL-7 through increased Tfh cell numbers in lymph nodes (13). It is possible that IL-7, in the presence of an exogenous antigen, and when provided under a limited time frame, may be useful for the induction of selected Tfh cell clones, which will enhance response to vaccination. It would be interesting to evaluate whether IL-7 provided in the context of vaccination leads to a transient upregulation of the B cell costimulatory molecules PD-1 and CD70 on Tfh cells and assess whether these molecules contribute to the development of memory B cells clones and PCs specific for vaccination antigens.

As reported in recent studies, Tfh cells may comprise heterogeneous populations of cells. In HIV-1-infected individuals, circulating IL-21+CD4+ T cells specific for different HIV-1 proteins provided a different quality of help to B cells (19). During chronic SIV infection, an expansion of Th1-biased Tfh cells was described to take place in lymphoid tissue; the Th1-biased Tfh cells had a phenotype and functions that distinguished them from conventional GC Tfh cells (44). It is not studied whether different subsets of Tfh cells may differ in CD127 expression and differentially regulate the expression of B-cell costimulatory molecules in response to IL-7 stimuli. In our study, Tfh cells were characterized by the expression of CXCR5; circulating CXCR5+CD4+ T cells were previously shown to be heterogeneous in the expression of several markers crucial for Tfh cell function and in their capacity to provide B cell help (15, 16). Additional studies are accordingly needed to address the expression and function of CD127 in relation to heterogeneity of CXCR5+CD4+ T cells.

In summary, attention should be paid in future studies to assess the role that IL-7 may have *in vivo* in dysregulating the expression of molecules on Tfh cells, which are pivotal for the generation of B cell responses within the GCs.

## Ethics Statement

The ethical committee at the Karolinska Institutet approved the study. Written consent was obtained from study participants.

## Author Contributions

FC designed the study and wrote the manuscript. RL, YB, and AN performed the experiments and wrote the manuscript.

## Conflict of Interest Statement

The authors declare that the research was conducted in the absence of any commercial or financial relationships that could be construed as a potential conflict of interest. The reviewer, VV, and handling Editor declared their shared affiliation, and the handling Editor states that the process nevertheless met the standards of a fair and objective review.
